# Effects of chitosan oligosaccharide on the growth performance, antioxidant capacity, immune function, intestinal digestive capacity and morphological structure in growing female minks

**DOI:** 10.1371/journal.pone.0332818

**Published:** 2025-09-19

**Authors:** Hongfei Liu, Wenli Li, Gaoqiang Fan, Qiaoyi Chen, Shulei Zhang, Beibei Zhang

**Affiliations:** College of Animal Science and Technology, Qingdao Agricultural University, Qingdao, Shandong Province, China; University of Life Sciences in Lublin, POLAND

## Abstract

This study aimed to investigate the effects of dietary chitosan oligosaccharide (COS) supplementation on growth performance, antioxidant capacity, immune function, duodenal digestive enzyme activity, and jejunal morphology in growing female minks. Ninety-six 12-week-old minks were randomly assigned to six groups (0, 100, 200, 300, 400, or 500 mg/kg COS), with 8 replicates per treatment and 2 minks per replicate, for an 8-week trial. The results showed that average daily gain (ADG) increased quadratically with increasing COS levels (*P* < 0.05) and 100 mg/kg COS significantly enhanced ADG (*P* < 0.05). Fresh pelt length increased linearly, and fresh pelt weight increased quadratically as COS levels increased (*P* < 0.05). COS supplementation significantly elevated serum immunoglobulin (Ig) A, IgG and complement 4 levels (*P* < 0.05). COS supplementation significantly increased serum superoxide dismutase activity and jejunal mucosal glutathione peroxidase activity, and significantly decreased serum malondialdehyde level (*P* < 0.05). Duodenal trypsin activity and the villus height-to-crypt depth ratio increased quadratically as COS levels increased (*P* < 0.05). In conclusion, dietary COS supplementation improved antioxidant capacity, immune function, duodenal digestive enzyme activity and jejunal morphology in growing female minks, thereby enhancing growth performance. The optimal dosage of COS is 100 mg/kg.

## Introduction

Mink (Neovison vison), a fur-bearing species of significant commercial importance, exhibits rapid growth and development during the growing period. The growth and development of the fur directly determine the economic value of minks. Hence, It is essential to ensure adequate nutrition in their diet during this stage [1]. Conventional mink feed containing animal by-products, viscera, and plant-derived ingredients are prone to spoilage and rancidity under summer high-temperature conditions, which may cause diarrhea and ultimately affecting the growth performance [2,3]. Therefore, developing effective nutritional strategies to maintain feed quality and support optimal growth becomes particularly important for mink production.

Chitosan oligosaccharide (COS) is an alkaline amino oligosaccharide that widely exists in nature and is categorized as a prebiotic compound. COS consists of 2–10 glucose units linked by β-1,4-glycosidic bonds, and it is the only natural cationic polysaccharide identified to date [4]. It possesses characteristics such as good water solubility, low molecular weight and high safety, making it widely applicable in the fields of biomedicine, food, and agriculture. Both COS and chitosan are derivatives of chitin, and COS can be produced by the degradation of chitosan. Compared with chitosan and chitin, COS exhibits a variety of biological activities such as antibacterial, antioxidant, and immunomodulatory effects [5]. Studies have demonstrated that COS can inhibit the reproduction of spoilage bacteria in fishery products mainly due to the positively charged COS molecules bind to negatively charged bacterial cell membranes, resulting in bacterial lysis [6].

Currently, some studies have demonstrated that dietary COS supplementation can improve animal growth performance, enhance antioxidant and immune function, and improve intestinal health. Gu et al. [7] found that dietary COS addition increased jejunal mucosal superoxide dismutase (SOD) and glutathione peroxidase (GSH-Px) activities while reducing jejunal interferon-gamma (IFN-γ) and interleukin-1 beta (IL-1β) levels in laying hens, effectively mitigating LPS-induced oxidative and immune stress. Osho et al. [8] demonstrated that dietary COS supplementation improved the jejunal morphology, enhanced ileal digestibility, and increased the activities of SOD, catalase (CAT), and GSH-Px in the jejunal mucosa of broiler chickens stimulated by dexamethasone, thereby improving growth performance. Zhou et al. [9] reported that the addition of COS in the diet of weaned piglets increased nutrient digestibility, reduced peripheral lymphocyte counts and diarrhea incidence, and enhanced growth performance. Cheng et al. [10] discovered that dietary COS addition significantly elevated serum levels of immunoglobulin A (IgA) and immunoglobulin G(IgG), enhanced the activities of CAT, GSH-Px, and SOD in Beagle dogs. Consequently, we infer that dietary supplementation with COS may confer beneficial effects on minks.

At present, there have been no reported studies on the dietary COS supplementation in growing female minks. Moreover, the optimal dosage of COS added to the diet of minks has not been determined yet. Therefore, this experiment aims to investigate the effects of different levels of COS supplementation in the diet on growth performance, fur quality, intestinal development, antioxidant capacity, immune function, duodenal digestive enzyme activity, and jejunal morphological structure in growing female minks. The findings will provide scientific basis for the application of COS in mink husbandry.

## Materials and methods

### Experimental design

The animal experiment was approved by the Animal Care and Use Committee of Qingdao Agricultural University (approval number DKY20240519). A total of 120 healthy female red-eyed white minks with similar initial body weights (940.93 ± 4.467) were randomly divided into 6 groups, with 10 replicates in each group and 2 minks in each replicate. The addition levels of COS in the diets of each group were 0 (control group), 100, 200, 300, 400, and 500 mg/kg respectively. The trial lasted for 8 weeks, divided into the first 4 weeks and the last 4 weeks. The ingredient composition and nutrient levels of the basal diet are shown in Table 1. The COS was provided by Qingdao XX Biotechnology Co., Ltd. (Qingdao, Shandong, China, purity 93.8%, deacetylation 91.2%, molecular weight ≤ 3000Da).

**Table 1:** Composition and nutrient levels of basal diets (air-dry basis) %.

Items	Week 1–4	Week 5–8
Extruded corn	5	5
Extruded soybean meal	3	3
Monkfish head	5	5
Cod Steak	10	10
Sea fishes	12	12
Pork Blood meal	1	0
Chicken frames	15	15
Chicken liver	8	8
Chicken head	15	15
Unhatched fertilized egg	23	23
Soybean oil	2	3
Premix^1^	1	1
Total	100	100
Nutrient level		
ME (MJ/kg) ^2^	17.93	17.98
Crude protein	36.79	33.25
Ether extract	23.89	25.37
Calcium	3.68	3.97
Phosphorus	0.94	0.90

^1^The Premix Provided the following Per kg of the diets: VA 6000 IU, VD3 540 IU, VE 37.5 mg, VK_3_ 0.72 mg, VB_1_ 11.4 mg, VB_2_ 7.2 mg, VB_6_ 3.6 mg, VB_12_ 19.5 μg, biotin 0.12 mg, folic acid 0.6 mg, nicotinic acid 14.7 mg, pantothenic acid 5.7 mg, VC 30 mg, Choline chloride 150 mg, Fe 30 mg, Cu 12.6 mg, Mn 15 mg, Zn 30 mg.

^2^ME was a calculated value, while the others were measured values.

### Animal management

The experiment was carried out from July to September 2024 at a mink farm. The minks had completed canine distemper and parvovirus vaccinations and have been implanted with melatonin. The minks were raised in sheds and fed twice in the morning and evening with free access to food and water, and the lighting throughout the experiment was natural light.

### Growth performance determination

The female minks were weighed at the beginning of the trial and the end of week 4 and week 8. The feed intake was accurately record for 3 consecutive days each week, and then the average daily gain (ADG), average daily feed intake (ADFI), and feed-to-gain ratio were calculated (F/G).

### Sample collection

At the end of the experiment, 8 minks were randomly selected from each group to measure the body length (from the tip of the nose to the base of the tail). Then, 10 mL of blood was collected from the heart in a coagulation-promoting tube and centrifuged at 3000 r/min for 10 minutes at 4°C.The serum was stored at −80°C for further analysis. After the minks were euthanized, the pelt was removed. The pelt weight and length (from the tip of the noe to the base of the tail) were measured. The mink was dissected, and all the intestines were separated. The intestinal length and weight were measured, and the ratio of intestinal length to body length was calculated. The duodenal digesta were placed in sterile 5 mL tubes and stored at −80°C for subsequent analysis. Segments of jejunum (1.5 cm in length) were collected and fixed in a 4% paraformaldehyde solution for histomorphological evaluation. Jejunum mucosa was scraped off with a sterile glass slide after rinsing with saline, frozen in liquid nitrogen, stored at −80°C for subsequent analysis.

### Serum immune indices determination

IgA, IgG, Immunoglobulin M(IgM), complement 3 (C3), and complement 4 (C4) were determined using ELISA kits (Shanghai Enzyme-linked Biotechnology Co., Ltd, China) according to the manufacturer’s protocols.

### Determination of antioxidant indicators in serum and jejunal mucosa

All the antioxidant indicator assay kits were purchased from Nanjing Jiancheng Bioengineering Institute (China). The Total Antioxidant Capacity (T-AOC) and the GSH-Px acitivities were determined using a colorimetric method. The activity of T-SOD was measured by the hydroxylamine method. Malondialdehyde (MDA) level was assessed using the thiobarbituric acid (TBA) method. Protein levels in jejunal mucosal homogenates were qualified using BCA assay kits (Cwbio, Beijing, China).

### Determination of duodenal digestive enzyme activity

The duodenal contents were prepared into a 10% (w/v) homogenate using physiological saline. Total protein contentration was determined using a BCA assay kits (Cwbio, Beijing, China). The activities of trypsin, lipase and amylase were measured using commerically available enzymatic assay kits (Nanjing Jiancheng Bioengineering Institute, China) following the munufacturer’s instructions.

### Jejunum morphology determination

The jejunal segments were embedded in paraffin to prepare paraffin sections. Hematoxylin-eosin (HE) staining was used to stain the tissue sections. For each slide, 10 intact, vertically oriented villi were selected for morphological examination under an optical microscope (Zeiss, Germany). Villus height (VH) and crypt depth (CD) were measured using ZEN imaging software (Carl Zeiss, Germany), and the villus height-to-crypt depth ratio (VCR) was calculated.

### Statistical analysis

All data were analyzed using one-way analysis of variance (ANOVA) followed by Duncan’s multiple range test in SPSS 26.0 software (IBM, USA). Linear and quadratic polynomial contrasts were performed to evaluate dose-dependent effects. The results were presented as means and standard errors of the mean (SEM). Differences were considered statistically significant at *P* < 0.05.

## Results

### Effects of COS on growth performance

As presented in Table 2, with the increasing level of COS supplementation, the ADG increased linearly and quadratically (**P* *< 0.05), and F/G decreased linearly (**P* *< 0.05) during the first 4 weeks. Compared with the control, 100 mg/kg COS significantly elevated final weight and ADG during the first 4 weeks (**P* *< 0.05). ADG increased quadratically as dietary COS increased during the entire 8-week (**P* *< 0.05). Compared with the control, 100 mg/kg COS significantly increased ADG during the entire 8-week (**P* *< 0.05). However, dietary COS addition had no significant effects on the ADG, ADFI and F/G of growing female minks during the last 4 weeks.

**Table 2:** Effects of dietary COS supplementation on growth performance in growing female minks (n = 8).

Items	COS supplementation levels (mg/kg)							*P*-value		
	0	100	200	300	400	500	SEM	ANOVA	Linear	Quadratic
BW (g)										
Week 4	1185.00^b^	1277.78^a^	1239.44^ab^	1208.89^b^	1229.00^ab^	1212.22^b^	8.585	0.043	0.808	0.094
Week 8	1288.33	1375.00	1324.50	1326.67	1342.00	1324.44	9.208	0.161	0.654	0.212
ADG (g)										
Week 1–4	9.54^bc^	12.14^a^	11.37^ab^	9.82^bc^	9.69^bc^	9.20^c^	0.280	0.006	0.043	0.024
Week 5–8	3.75	3.79	4.40	3.89	4.06	3.82	0.148	0.852	0.824	0.360
Week 1–8	6.40^b^	7.75^a^	7.22^ab^	7.01^ab^	6.94^ab^	6.62^b^	0.122	0.016	0.501	0.013
ADFI (g)										
Week 1–4	145.76	156.91	154.85	148.68	148.25	144.23	1.593	0.117	0.203	0.049
Week 5–8	136.23	142.80	141.59	132.51	141.63	137.33	1.478	0.324	0.812	0.692
Week 1–8	141.00	148.62	147.23	142.48	146.50	145.90	0.956	0.129	0.474	0.351
F/G										
Week 1–4	14.82^abc^	13.48^c^	14.05^bc^	15.31^abc^	16.09^ab^	16.25^a^	0.303	0.036	0.007	0.199
Week 5–8	37.27	36.87	35.64	34.36	34.54	35.00	1.188	0.973	0.441	0.723
Week 1–8	21.84	19.34	20.61	20.56	21.60	21.64	0.304	0.112	0.340	0.083

BW, body weight; ADG, average daily gain; ADFI, average daily feed intake; F/G, the ratio of feed to gain.

Means in the same row with different superscripts are significantly different (**P* *< 0.05).

### Effects of COS on fur and intestinal development

Table 3 illustrated that as the dietary level of COS increased, there was a significant linear increase in fresh pelt length and intestine length-to- body length ratio (**P* *< 0.05). Meanwhile, the fresh pelt weight exhibited a significant quadratic increase (**P* *< 0.05). Compared with the control, 100 ~ 500 mg/kg COS significantly increased fresh pelt length (*P* < 0.05). Additionally, 100 and 300 mg/kg COS significantly increased fresh pelt weight (*P* < 0.05). Moreover, 100, 300, 400 and 500 mg/kg COS significantly elevated intestinal length-to-body length ratio (**P* *< 0.05).

**Table 3:** Effects of dietary COS supplementation on fur and intestinal development in growing female minks (n = 8).

Items	COS supplementation levels (mg/kg)							*P*-value		
	0	100	200	300	400	500	SEM	ANOVA	Linear	Quadratic
Body length (cm)	40.12	40.57	41.10	40.12	40.08	40.25	0.214	0.741	0.689	0.465
Fresh pelt length (cm)	42.54^b^	45.89^a^	45.76^a^	46.16^a^	45.86^a^	46.40^a^	0.368	0.035	0.009	0.061
Fresh pelt weight (kg)	0.40^c^	0.47^a^	0.44^abc^	0.45^ab^	0.42^bc^	0.42^bc^	0.007	0.029	0.697	0.010
Intestinal length (cm)	159.33^c^	173.14^a^	162.25^bc^	170.50^a^	170.00^a^	168.00^ab^	1.202	0.005	0.063	0.122
Intestinal weight (g)	44.30	46.90	45.13	43.97	45.46	44.09	0.529	0.620	0.566	0.584
Intestine length-to- body length ratio	3.89^b^	4.36^a^	3.95^b^	4.26^a^	4.25^a^	4.18^a^	0.040	0.001	0.039	0.136

Means in the same row with different superscripts are significantly different (**P* *< 0.05).

### Effects of COS on serum immune indices

According to Table 4, compared to the control, 100 mg/kg COS significantly increased the serum IgA level (**P* *< 0.05), while 100 and 200 mg/kg COS significantly increased the IgG level (**P* *< 0.05). Serum C4 level was increased linearly and quadratically as dietary COS increased. The supplementation of 100–500 mg/kg COS resulted in a significant increase in serum C4 levels compared to the control (**P* *< 0.05).

**Table 4:** Effects of dietary COS supplementation on serum immune indices in growing female minks (n = 8).

Items	COS supplementation levels/(mg/kg)							*P*-value		
	0	100	200	300	400	500	SEM	ANOVA	Linear	Quadratic
IgA(μg/mL)	651.59^b^	733.28^a^	680.08^b^	665.24^b^	665.29^b^	662.61^b^	7.412	0.014	0.229	0.163
IgM(μg/mL)	1574.84	1713.44	1659.45	1632.26	1609.73	1606.96	17.978	0.306	0.627	0.153
IgG(g/L)	16.48^c^	20.15^a^	18.49^ab^	18.12^bc^	18.08^bc^	18.61^ab^	0.296	0.012	0.447	0.121
C3(μg/mL)	112.69	118.42	119.03	116.07	115.89	116.31	1.304	0.803	0.786	0.334
C4(μg/mL)	96.78^b^	102.32^a^	103.48^a^	103.42^a^	102.52^a^	101.68^a^	0.667	0.025	0.046	0.005

IgA, immunoglobulin A; IgM, immunoglobulin M; IgG, immunoglobulin G.C3, complement 3; C4, complement 4.

Means in the same row with different superscripts are significantly different (**P* *< 0.05).

### Effects of COS on the serum antioxidant capacity

As listed in Table 5, dietary supplementation with 100 and 200 mg/kg COS significantly enhanced serum SOD activity compared to the control group (**P* *< 0.05). Serum MDA level demonstrated a quadratic response as dietary COS increased (**P* *< 0.05), with 100 ~ 500 mg/kg COS significantly reducing serum MDA level relative to the control (**P* *< 0.05).

**Table 5:** Effects of dietary COS supplementation on serum antioxidant capacity in growing female minks (n = 8).

Items	COS supplementation levels/(mg/kg)							*P*-value		
	0	100	200	300	400	500	SEM	ANOVA	Linear	Quadratic
T-AOC(U/mL)	5.23	5.50	5.63	5.38	5.44	5.35	0.183	0.995	0.954	0.640
T-SOD(U/mL)	53.43^c^	63.08^a^	61.55^ab^	55.69^bc^	57.55^abc^	54.76^bc^	1.039	0.023	0.416	0.062
GSH-Px(U/mL)	5788.71	6311.66	6342.76	6248.43	6153.66	6087.24	81.966	0.398	0.583	0.065
MDA (nmol/mL)	17.53^a^	11.38^b^	11.28^b^	12.86^b^	13.08^b^	13.59^b^	0.548	0.004	0.176	0.002

T-AOC, total antioxidant capacity; T-SOD, total superoxide dismutase; GSH-Px, glutathione peroxidase;

MDA, malondialehyde.

Means in the same row with different superscripts are significantly different (**P* *< 0.05).

### Effects of COS on duodenal digestive enzyme activities

As shown in Table 6, trypsin activity in duodenum increased quadratically with increased dietary COS concentration (**P* *< 0.05). Compared to the control 100,200,300 and 500 mg/kg COS significantly enhanced trypsin activity in duodenum (*P* < 0.05). No significance was observed in duodenal lipase and amylase (**P* *> 0.05).

**Table 6:** Effects of dietary COS supplementation on duodenal digestive enzyme activities in growing female minks (n = 8).

Items	COS supplementation levels/(mg/kg)							*P*-value		
	0	100	200	300	400	500	SEM	ANOVA	Linear	Quadratic
Trypsin (U/mg prot)	784.19^b^	1191.53^a^	1117.62^a^	1241.74^a^	962.53^ab^	1090.93^a^	41.791	0.021	0.216	0.013
Lipase (U/g prot)	12.06	10.44	11.17	12.29	10.90	11.82	0.604	0.955	0.921	0.769
Amylase (U/mg prot)	2.68	2.80	2.72	2.58	2.60	2.69	0.050	0.847	0.534	0.862

Means in the same row with different superscripts are significantly different (**P* *< 0.05).

### Effects of COS on jejunal morphology

**Fig 1:**
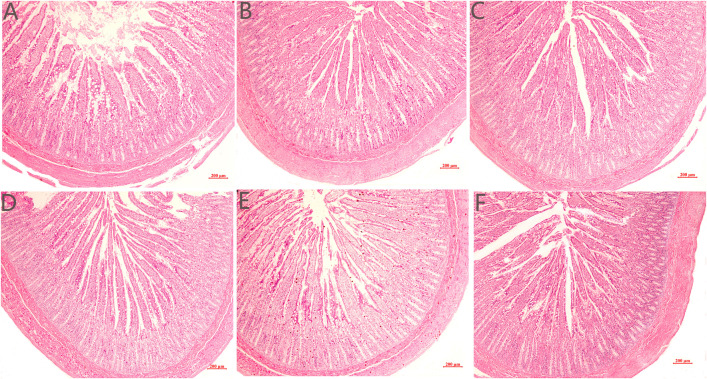
Effect of COS supplementation on the jejunal morphology in growing female minks.

As presented in Fig 1, The jejunal villi in the control group were short and thick, with mild villus tip loss and lymphocyte infiltration. The villi in the COS-treated groups were long and slender, and exhibited regular arrangement and relatively intact morphology. As shown in Table 7, compared with the control, 200 mg/kg COS significantly elevated villus height in the jejunum (**P* *< 0.05). The VCR in the jejunum increased quadratically as dietary COS increased (**P* *< 0.05). Compared with the control, 100–200 mg/kg COS significantly increased jejunal VCR (**P* *< 0.05).

**Table 7:** Effects of dietary COS supplementation on jejunal morphology in growing female minks (n = 8).

Items	COS supplementation levels/(mg/kg)								*P*-value	
	0	100	200	300	400	500	SEM	ANOVA	Linear	Quadratic
Villus Height (μm)	929.44^b^	1011.64^ab^	1051.28^a^	948.91^b^	937.48^b^	980.12^ab^	12.000	0.014	0.748	0.104
Crypt Depth (μm)	429.26	414.95	415.07	416.55	413.12	412.18	1.810	0.065	0.014	0.175
VCR	2.17^c^	2.44^ab^	2.53^a^	2.28^bc^	2.27^bc^	2.33^bc^	0.030	0.003	0.874	0.017

VCR, the ratio of villus height to crypt depth.

Means in the same row with different superscripts are significantly different (**P* *< 0.05).

### Effects of COS on the antioxidant capacity of jejunal mucosa

As listed in Table 8, GSH-Px activity in the jejunal mucosa increased linearly and quadratically with increasing levels of COS (**P* *< 0.05). Compared with the control, 100 ~ 500 mg/kg COS significantly increased GSH-Px activity in the jejunal mucosa (**P* *< 0.05). No significance was observed in T-AOC and T-SOD activities and MDA level in the jejunal mucosa (**P* *> 0.05).

**Table 8:** Effects of dietary COS supplementation on jejunal mucosal antioxidant capacity in growing female minks (n = 8).

Items	COS supplementation levels/(mg/kg)							*P*-value		
	0	100	200	300	400	500	SEM	ANOVA	Linear	Quadratic
T-AOC (U/mg prot)	1.19	1.49	1.32	1.33	1.36	1.36	0.058	0.809	0.704	0.814
T-SOD (U/mg prot)	44.51	50.26	56.37	45.78	48.40	50.25	1.739	0.424	0.730	0.396
GSH-Px (U/mg prot)	18.31^b^	25.45^a^	27.15^a^	26.21^a^	26.51^a^	25.41^a^	0.846	0.020	0.021	0.010
MDA (nmol/mg prot)	1.31	1.02	1.05	1.03	1.24	1.22	0.041	0.151	0.792	0.024

T-AOC, total antioxidant capacity; T-SOD, total superoxide dismutase; GSH-Px, glutathione peroxidase;

MDA, malondialehyde.

Means in the same row with different superscripts are significantly different (**P* *< 0.05).

## Discussion

### Growth performance

Our results showed that dietary COS supplementation significantly increased the final body weight at week 4 and ADG during the first 4 weeks and entire 8 weeks in growing female minks, with the best effect observed at an addition of 100 mg/kg COS. Lan et al. [11] found that the body weights of frizzled chickens increased with the addition of COS, and the supplementation of 600 and 900 mg/kg COS in the diet significantly reduced the feed-to-gain ratio. Li et al. [12] discovered that dietary supplementation of 30 mg/kg COS improved the growth performance, breast meat quality, and oxidative status of broiler chickens. However, Ummay et al. [13] found that the dietary addition of 350 mg/kg COS had no significant effect on the growth performance of broiler chickens. Additionally, Wei et al. [14] shown that the supplementation of 200 mg/kg COS in the diet did not significantly influence the growth performance of blue foxes. But a smoother and lustrousan fur was observed. The varying research results on the effects of COS on growth performance may be attributed to differences in the degree of deacetylation, molecular weight of COS, and the experimental animals used. The lack of significant growth performance changes in growing female minks during the last 4 weeks may be due to the high summer temperature, which decreased feed intake.

Although COS has been reported to reduce lipid absorption by binding to fat, fatty acids and bile acids, thereby inducing weight loss in obese models or high-fat-diet studies [15–17], our results demonstrated a significant growth-promoting effect in minks. This apparent discrepancy can be explained by the following reasons: Firstly, the dosage used in our experiment (100–500 mg/kg) was likely insufficient to significantly impair fat absorption. Secondly, minks are carnivores adapted to a high-fat diet. The benefits of COS, notably its role in improving immunity, antioxidant status and intestinal health, clearly outweighed any potential reduction in lipid absorption, ultimately leading to a net gain in growth performance.

### Fur and intestinal development

The economic value of mink depends on the quality of the pelts [18]. In this study, dietary COS supplementation increased the fresh pelt length and fresh pelt weight of growing female minks, indicating its positive effect on the development of the fur. Research has shown that COS can stimulate fibroblast proliferation by regulating the production of transforming growth factors, thereby increasing the levels of collagen and promoting the development of connective tissues [19]. This may be the reason for its ability to improve the fresh pelt length and weight. In addition, we found that COS significantly increased the total intestinal length of growing female minks, indicating that COS promoted intestinal development. A longer intestine can enhance the retention time of feed, thereby promoting nutrient digestion and absorption [20]. Similarly, Lan et al. [21] reported that dietary COS supplementation significantly increased the relative length and relative weight of the duodenum, as well as the relative weight of the ileum in broiler chickens.

### Immune indices

Serum immunoglobulins and complement components play important roles in enhancing immune function and clearing pathogens [22,23]. Youssef et al. [24] demonstrated that dietary COS supplementation increased the serum IgG and IgM levels of laying hens. Jahan et al. [25] showed that COS administration in the diet elevated the serum IgM level in lambs. In addition, Duan et al. [26] observed increased serum IgG and C3 levels in sows fed COS-supplemented diets. According to Zhao et al. [27], serum C4 level was elevated in weaned piglets receiving COS. In the present study, dietary COS supplementation increased IgA, IgG and C4 levels in growing female minks, indicating that COS enhanced the immune function of minks. Previous research has shown that COS promoted the differentiation of T cells and B cells by stimulating macrophages to secrete cytokines, thereby facilitating immunoglobulin production [28]. This may be the reason for the enhanced immunoglobulin in minks.

### Antioxidant capacity

T-SOD, T-AOC, MDA and GSH-Px are critical indicators closely related to redox homeostasis in organisms. T-SOD is a key enzyme responsible for scavenging reactive oxygen species [29]. MDA, a product of lipid peroxidation, serves as an indicator of oxidative damage, with its level reflecting the degree of cellylar damage [30]. GSH-Px is a vital antioxidant enzyme that eliminating hydrogen peroxide and organic peroxides, thereby protecting cells from oxidative stress [31]. Lan et al. [32] found that dietary supplementation of COS increased SOD and CAT activities and reduced the MDA level in the muscle of broiler chickens under transportation stress. Chang et al. [33] demonstrated that COS reduced the MDA level and enhanced SOD and GSH-Px activities in the muscle tissue of yellow-feathered broiler chickens under heat stress. Fathi et al. [34] reported that dietary supplementation of COS elevated T-SOD, CAT, and GSH-Px activities and reduced MDA level in both serum and liver of broiler chickens under cold stress. The present study showed that dietary COS supplementation increased the serum T-SOD activity and jejunal mocosa GSH – Px activity and reduced serum MDA level, indicating the antioxidant capacity of COS. Some studies have shown that the molecular structure of COS contains a large number of amino and hydroxyl groups, which can effectively scavenge free radicals in the body [35]. Furthermore, the loose structure and short molecular chain of COS reduce its ability to form intramolecular hydrogen bonds. This results in higher reactivity of the amino and hydroxyl groups, thereby enhancing its capacity to eliminate free radicals [36].

### Digestive enzyme activities

The activities of digestive enzymes reflect the functional status of the digestive system and serve as important indicators of intestinal health [37]. Trypsin, lipase and amylase are crucial for the absorption of nutrients [38]. Trypsin cleaves proteins into smaller polypeptides, facilitating their subsequent breakdown into amino acids by other proteases [39]. Lipase catalyzes the hydrolysis of triglycerides into glycerol and free fatty acids, providing energy for animals [40]. Amylase degrades starch and glycogen into smaller carbohydrate molecules [41]. Zhang et al. [42] found that the dietary supplementation of COS increased the activities of protease, lipase and amylase in the intestine of loach. Moreover, Ramasamy et al. [43] observed increased protease, lipase and amylase activities in the intestine of silver carp receiving COS. In addition, Wang et al. [44] showed that the supplementation of COS in the feed of sows during the late pregnancy and lactation periods increased the trypsin and lactase activities in the jejunum of 21-day-old piglets. Su et al. [45] found that the dietary COS supplementation significantly enhances the activities of protease and lipase in the intestines of tiger puffer. In this study, dietary COS supplementation increased the duodenal trypsin activity in minks, indicating improved protein digestive capacity. The reason may be due to COS’s unique properties as a basic amino oligosaccharide with a positively charged amino group, which enables it to bind excess hydrogen ions and help maintain a stable, weakly alkaline duodenal environment optimal for trypsin and other proteases.

### Intestinal morphology

The intestine is the main site for the absorption of nutrients. Increased villus height improves intestinal nutrient utilization, while decreased crypt depth indicates higher cell maturity and enhanced secretory function, with the VCR serving as a well-established biomarker reflecting intestinal health status [46–48]. Li et al. [49] reported that dietary COS supplementation reduced the crypt depth in the jejunum, increased the villus height in the duodenum, and increased the VCR in both the duodenum and jejunum of broiler chickens. Zhao et al. [50] discovered that the dietary COS addition increased jejunal villus height and villus surface area and decreased ileal VCR in weaned squabs. Manrong et al. [51] demonstrated the dietary supplementation of COS increased the villus height and VCR in the jejunum of piglets challenged with Escherichia coli. Additionally, Thongsong et al. [52] found that the dietary supplementation of COS significantly enhances the villus height and VCR in the duodenum, jejunum, and ileum of weaned piglets. In this study, the addition of COS significantly increased the jejunal villus height and VCR. Moreover, histomorphological images also demonstrated superior villus and crypt architectures in the COS-treated groups, indicating that COS can improve the intestinal morphology in growing female minks. This beneficial effect may be attributed to N-acetylglucosamine, the primary component of COS, which can bind to specific pathogenic bacteria and inhibit their colonization in intestinal tissues, thereby enhancing intestinal morphology [53,54].

## Conclusions

Dietary COS supplementation can enhance the immune function and antioxidant capacity, improve the intestinal morphology, increase digestive enzyme activity, and enhance the growth performance of growing female minks. The recommended dietary supplementation level of COS for growing female minks is 100 mg/kg.

## Supporting information

S1 Data Raw Data. (XLSX)

## Abbreviations

COS: chitosan oligosaccharide
ADG: average daily gain
ADFI: average daily feed intake
F/G: feed-to-gain
IgA: immunoglobulin A
IgM: immunoglobulin M
IgG: immunoglobulin G
C3: complement 3
C4: complement 4
T-AOC: total antioxidant capacity
MDA: malondialdehyde
TBA: thiobarbituric acid
T-SOD: total superoxide dismutase
GSH-Px: glutathione peroxidase
VCR: villus height-to-crypt depth ratio.

## References

[1] Zhang N-Z, Li W-H, Yu H-J, Liu Y-J, Qin H-T, Jia W-Z, et al. Novel study on the prevalence of Trichinella spiralis in farmed American minks (Neovison vison) associated with exposure to wild rats (Rattus norvegicus) in China. Zoonoses Public Health. 2022;69(8):938–43. doi: 10.1111/zph.12991 36345967

[2] Mathiesen R, Birch JM, Chriél M, Jensen HE, Agger JF, Heegaard PMH, et al. Mink (Neovison vison) kits with pre-weaning diarrhea have elevated serum amyloid A levels and intestinal pathomorphological similarities with New Neonatal Porcine Diarrhea Syndrome. Acta Vet Scand. 2018;60(1):48. doi: 10.1186/s13028-018-0403-7 30111375 PMC6094914

[3] Lyhs U, Frandsen H, Andersen B, Nonnemann B, Hjulsager C, Pedersen K, et al. Microbiological quality of mink feed raw materials and feed production area. Acta Vet Scand. 2019;61(1):56. doi: 10.1186/s13028-019-0489-6 31752948 PMC6873557

[4] Kamal M, Zhu L, Abd El-Hack ME, Arif M, Li F, Cheng Y. Functional roles of mannan and chitosan oligosaccharides on animal health and nutrition: A review. Carbohydrate Polymer Technologies and Applications. 2025;10:100764. doi: 10.1016/j.carpta.2025.100764

[5] Guan Z, Feng Q. Chitosan and Chitooligosaccharide: The Promising Non-Plant-Derived Prebiotics with Multiple Biological Activities. Int J Mol Sci. 2022;23(12):6761. doi: 10.3390/ijms23126761 35743209 PMC9223384

[6] Zhang L, Wang Y, Ling S, Yuan M, Sun Q, Dong X. Antibacterial mechanism of chitooligosaccharides against specific spoilage organisms in chilled processed fish paste products. Food Control. 2025;172:111148. doi: 10.1016/j.foodcont.2025.111148

[7] Gu YF, Chen YP, Jin R, Wang C, Wen C, Zhou YM. Dietary chitooligosaccharide supplementation alleviates intestinal barrier damage, and oxidative and immunological stress in lipopolysaccharide-challenged laying hens. Poult Sci. 2022;101(4):101701. doi: 10.1016/j.psj.2022.101701 35150943 PMC8844238

[8] Osho SO, Adeola O. Chitosan oligosaccharide supplementation alleviates stress stimulated by in-feed dexamethasone in broiler chickens. Poult Sci. 2020;99(4):2061–7. doi: 10.1016/j.psj.2019.11.047 32241491 PMC7587614

[9] Zhou TX, Cho JH, Kim IH. Effects of supplementation of chito-oligosaccharide on the growth performance, nutrient digestibility, blood characteristics and appearance of diarrhea in weanling pigs. Livestock Science. 2012;144(3):263–8. doi: 10.1016/j.livsci.2011.12.009

[10] Cheng G, Hu T, Zeng Y, Yan L, Liu Y, Wang Y, et al. Enhancing immune response, antioxidant capacity, and gut health in growing beagles through a chitooligosaccharide diet. Front Vet Sci. 2024;10:1283248. doi: 10.3389/fvets.2023.1283248 38274661 PMC10808298

[11] Lan R, Chen X, Zhang Y, Luo H. Effects of dietary chitosan oligosaccharides supplementation on meat quality, chemical composition and anti-oxidant capacity in frizzled chickens. Italian Journal of Animal Science. 2023;22(1):639–50. doi: 10.1080/1828051x.2023.2228826

[12] Li J, Wang S, Chen Y, Cheng Y, Wen C, Zhou Y. Dietary chitooligosaccharide supplementation improves mineral deposition, meat quality and intramuscular oxidant status in broilers. J Sci Food Agric. 2023;103(2):764–9. doi: 10.1002/jsfa.12187 36054497

[13] Ayman U, Akter L, Islam R, Bhakta S, Rahman MdA, Islam MR, et al. Dietary chitosan oligosaccharides improves health status in broilers for safe poultry meat production. Annals of Agricultural Sciences. 2022;67(1):90–8. doi: 10.1016/j.aoas.2022.05.003

[14] Wei J, Su J, Wang G, Li W, Wen Z, Liu H. Chitooligosaccharides improves intestinal mucosal immunity and intestinal microbiota in blue foxes. Front Immunol. 2024;15:1506991. doi: 10.3389/fimmu.2024.1506991 39628477 PMC11611864

[15] Zhang J, Zhang W, Mamadouba B, Xia W. A comparative study on hypolipidemic activities of high and low molecular weight chitosan in rats. Int J Biol Macromol. 2012;51(4):504–8. doi: 10.1016/j.ijbiomac.2012.06.018 22728057

[16] Wang J, He W, Yang D, Cao H, Bai Y, Guo J, et al. Beneficial Metabolic Effects of Chitosan and Chitosan Oligosaccharide on Epididymal WAT Browning and Thermogenesis in Obese Rats. Molecules. 2019;24(24):4455. doi: 10.3390/molecules24244455 31817377 PMC6943480

[17] Lee J-Y, Kim TY, Kang H, Oh J, Park JW, Kim S-C, et al. Anti-Obesity and Anti-Adipogenic Effects of Chitosan Oligosaccharide (GO2KA1) in SD Rats and in 3T3-L1 Preadipocytes Models. Molecules. 2021;26(2):331. doi: 10.3390/molecules26020331 33440605 PMC7827767

[18] Wang L, Zhou S, Liu G, Lyu T, Shi L, Dong Y, et al. The Mechanisms of Fur Development and Color Formation in American Mink Revealed Using Comparative Transcriptomics. Animals (Basel). 2022;12(22):3088. doi: 10.3390/ani12223088 36428316 PMC9686883

[19] Cheng G, Zeng Y, Wang Y, Xia J, Lei X, Chen D, et al. Effect of dietary supplementation with chitooligosaccharides on wound healing in dogs. Veterinary Medicine & Sci. 2024;10(2). doi: 10.1002/vms3.1359

[20] Duque-Correa MJ, Clauss M, Meloro C, Abraham AJ. Does intestine length explain digesta retention times in birds and mammals?. Comp Biochem Physiol A Mol Integr Physiol. 2025;300:111789. doi: 10.1016/j.cbpa.2024.111789 39653272

[21] Lan R, Wu F, Wang Y, Lin Z, Wang H, Zhang J, et al. Chitosan oligosaccharide improves intestinal function by promoting intestinal development, alleviating intestinal inflammatory response, and enhancing antioxidant capacity in broilers aged d 1 to 14. Poult Sci. 2024;103(2):103381. doi: 10.1016/j.psj.2023.103381 38157786 PMC10790092

[22] Xu L, Wei Z, Guo Y, Guo B, Cai L, Yan J, et al. Effects of dietary supplementation with fermented flaxseed meal on the growth performance, immune function, and intestinal microbiota of growing pigs. Animal Feed Science and Technology. 2024;316:116079. doi: 10.1016/j.anifeedsci.2024.116079

[23] Bai H, Mu L, Qiu L, Chen N, Li J, Zeng Q, et al. Complement C3 Regulates Inflammatory Response and Monocyte/Macrophage Phagocytosis of Streptococcus agalactiae in a Teleost Fish. Int J Mol Sci. 2022;23(24):15586. doi: 10.3390/ijms232415586 36555227 PMC9779060

[24] Youssef IM, Khalil HA, Shakoori AM, Bagadood RM, Alyahyawi AY, Alhazzaa RA, et al. Immune response, hematological traits, biochemical blood parameters, and histological status of laying hens influenced by dietary chitosan-oligosaccharides. Poult Sci. 2023;102(9):102834. doi: 10.1016/j.psj.2023.102834 37390556 PMC10466251

[25] Jahan M, Wilson C, McGrath S, Francis N, Wynn PC, Du Y, et al. Chitosan Oligosaccharide Supplementation Affects Immunity Markers in Ewes and Lambs during Gestation and Lactation. Animals (Basel). 2022;12(19):2609. doi: 10.3390/ani12192609 36230349 PMC9558557

[26] Duan X, Tian G, Chen D, Yang J, Zhang L, Li B, et al. Effects of diet chitosan oligosaccharide on performance and immune response of sows and their offspring. Livestock Science. 2020;239:104114. doi: 10.1016/j.livsci.2020.104114

[27] Zhao P, Piao X, Zeng Z, Li P, Xu X, Wang H. Effect of Forsythia suspensa extract and chito-oligosaccharide alone or in combination on performance, intestinal barrier function, antioxidant capacity and immune characteristics of weaned piglets. Anim Sci J. 2017;88(6):854–62. doi: 10.1111/asj.12656 27758020

[28] Dang Y, Li S, Wang W, Wang S, Zou M, Guo Y, et al. The effects of chitosan oligosaccharide on the activation of murine spleen CD11c+ dendritic cells via Toll-like receptor 4. Carbohydrate Polymers. 2011;83(3):1075–81. doi: 10.1016/j.carbpol.2010.08.071

[29] Zheng L, Liu Y, Zhang Y, Xu B, Sagada G, Wang Z, et al. Comparative study on the effects of crystalline L-methionine and methionine hydroxy analogue calcium supplementations in the diet of juvenile Pacific white shrimp (Litopenaeus vannamei). Front Physiol. 2023;14:1067354. doi: 10.3389/fphys.2023.1067354 36793420 PMC9923173

[30] Liao G, Li J, Yu J, Wang W, Liu H, Zhang Z, et al. Dietary Bacillus subtilis HGcc-1 improves the growth performance, α-amylase and lipase activities, immunity and antioxidant capacity, intestinal microbiota, and heat stress resistance in Pacific white shrimp (Litopenaeus vannamei). Int J Biol Macromol. 2025;291:138987. doi: 10.1016/j.ijbiomac.2024.138987 39706398

[31] Lin W, Hou J, Guo H, Li L, Wang L, Zhang D, et al. The synergistic effects of waterborne microcystin-LR and nitrite on hepatic pathological damage, lipid peroxidation and antioxidant responses of male zebrafish. Environ Pollut. 2018;235:197–206. doi: 10.1016/j.envpol.2017.12.059 29289830

[32] Lan R, Chang Q, Lu Y. Effects of chitosan oligosaccharides on meat quality, muscle energy metabolism and anti-oxidant status in broilers that have experienced transport stress. Anim Prod Sci. 2021;61(15):1625–32. doi: 10.1071/an20249

[33] Chang Q, Lu Y, Lan R. Chitosan oligosaccharide as an effective feed additive to maintain growth performance, meat quality, muscle glycolytic metabolism, and oxidative status in yellow-feather broilers under heat stress. Poult Sci. 2020;99(10):4824–31. doi: 10.1016/j.psj.2020.06.071 32988519 PMC7598338

[34] Fathi M, Saeidian S, Baghaeifar Z, Varzandeh S. Chitosan oligosaccharides in the diet of broiler chickens under cold stress had anti-oxidant and anti-inflammatory effects and improved hematological and biochemical indices, cardiac index, and growth performance. Livestock Science. 2023;276:105338. doi: 10.1016/j.livsci.2023.105338

[35] Sahariah P, Másson M. Antimicrobial Chitosan and Chitosan Derivatives: A Review of the Structure-Activity Relationship. Biomacromolecules. 2017;18(11):3846–68. doi: 10.1021/acs.biomac.7b01058 28933147

[36] Wen F, Liu Y, Yang H, Yan X, Zhang Y, Zhong Z. Preparation, characterization, antioxidant, and antifungal activity of phenyl/indolyl-acyl chitooligosaccharides. Carbohydr Res. 2024;538:109077. doi: 10.1016/j.carres.2024.109077 38479043

[37] Zhang L, Wang Y, Zhang R, Jia H, Liu X, Zhu Z. Effects of three probiotics and their interactions on the growth performance of and nutrient absorption in broilers. PeerJ. 2022;10:e13308. doi: 10.7717/peerj.13308 35602903 PMC9121878

[38] Hakim AH, Zulkifli I, Farjam AS, Awad EA, Ramiah SK. Impact of Feeding Fermented Palm Kernel Cake and High Dietary Fat on Nutrient Digestibility, Enzyme Activity, Intestinal Morphology and Intestinal Nutrient Transporters mRNA Expression in Broiler Chickens under Hot and Humid Conditions. Animals (Basel). 2022;12(7):882. doi: 10.3390/ani12070882 35405871 PMC8997065

[39] Nuss AB, Gulia-Nuss M. Trypsin, the Major Proteolytic Enzyme for Blood Digestion in the Mosquito Midgut. Cold Spring Harb Protoc. 2023;2023(4):pdb.top107656. doi: 10.1101/pdb.top107656 36787964

[40] Thakur SJ. Lipases, its sources, properties and applications: a review. Int J Sci Eng Res. 2012;3:1–29.

[41] Hasan MT, Kim HJ, Hur S-W, Jeong S-M, Kim K-W, Lee S. Dietary Exogenous α-Amylase Modulates the Nutrient Digestibility, Digestive Enzyme Activity, Growth-Related Gene Expression, and Diet Degradation Rate of Olive Flounder (Paralichthys olivaceus). J Microbiol Biotechnol. 2023;33(10):1390–401. doi: 10.4014/jmb.2303.03033 37463868 PMC10619548

[42] Zhang B. Dietary chitosan oligosaccharides modulate the growth, intestine digestive enzymes, body composition and nonspecific immunity of loach Paramisgurnus dabryanus. Fish Shellfish Immunol. 2019;88:359–63. doi: 10.1016/j.fsi.2019.03.006 30851451

[43] Harikrishnan R, Devi G, Van Doan H, Gatphayak K, Balasundaram C, El-Haroun E, et al. Immunomulation effect of alginic acid and chitooligosaccharides in silver carp (Hypophthalmichthys molitrix). Fish Shellfish Immunol. 2022;128:592–603. doi: 10.1016/j.fsi.2022.08.009 35977648

[44] Wang X, Fang T, Chen D, Pu J, Tian G, He J, et al. Maternal chitosan oligosaccharide supplementation during late gestation and lactation optimizes placental function in sows and intestinal function in 21-day-old IUGR suckling piglets. Front Vet Sci. 2024;11:1463707. doi: 10.3389/fvets.2024.1463707 39606660 PMC11600973

[45] Su P, Han Y, Jiang C, Ma Y, Pan J, Liu S, et al. Effects of chitosan-oligosaccharides on growth performance, digestive enzyme and intestinal bacterial flora of tiger puffer (Takifugu rubripesTemminck et Schlegel, 1850). J Appl Ichthyol. 2017;33(3):458–67. doi: 10.1111/jai.13282

[46] Santos RR, Ooosterveer-van der Doelen MAM, Tersteeg-Zijderveld MHG, Molist F, Gehring R. Induction of gut leakage in young broiler chickens fed a diet with low rye inclusion. Heliyon. 2021;7(12):e08547. doi: 10.1016/j.heliyon.2021.e08547 34917817 PMC8665344

[47] Su G, Wang L, Zhou X, Wu X, Chen D, Yu B, et al. Effects of essential oil on growth performance, digestibility, immunity, and intestinal health in broilers. Poult Sci. 2021;100(8):101242. doi: 10.1016/j.psj.2021.101242 34174571 PMC8242051

[48] Zhang Z, Zhu T, Zhang L, Xing Y, Yan Z, Li Q. Critical influence of cytokines and immune cells in autoimmune gastritis. Autoimmunity. 2023;56(1):2174531. doi: 10.1080/08916934.2023.2174531 36762543

[49] Li J, Cheng Y, Chen Y, Qu H, Zhao Y, Wen C, et al. Dietary Chitooligosaccharide Inclusion as an Alternative to Antibiotics Improves Intestinal Morphology, Barrier Function, Antioxidant Capacity, and Immunity of Broilers at Early Age. Animals (Basel). 2019;9(8):493. doi: 10.3390/ani9080493 31357589 PMC6719223

[50] Zhao W, Jia Y, Li R, Li J, Zou X, Dong X. Effects of dietary Chitosan oligosaccharides supplementation on Th17/Treg balance and gut microbiota of early weaned pigeon squabs. Poult Sci. 2024;103(10):104088. doi: 10.1016/j.psj.2024.104088 39067116 PMC11338107

[51] Yu M, Meng T, He W, Huang H, Liu C, Fu X, et al. Dietary Chito-oligosaccharides Improve Intestinal Immunity via Regulating Microbiota and Th17/Treg Balance-Related Immune Signaling in Piglets Challenged by Enterotoxigenic E. coli. J Agric Food Chem. 2021;69(50):15195–207. doi: 10.1021/acs.jafc.1c06029 34881888

[52] Thongsong B, Suthongsa S, Pichyangkura R, Kalandakanond-Thongsong S. Effects of chito-oligosaccharide supplementation with low or medium molecular weight and high degree of deacetylation on growth performance, nutrient digestibility and small intestinal morphology in weaned pigs. Livestock Science. 2018;209:60–6. doi: 10.1016/j.livsci.2018.01.011

[53] Ofek I, Hasty DL, Sharon N. Anti-adhesion therapy of bacterial diseases: prospects and problems. FEMS Immunol Med Microbiol. 2003;38(3):181–91. doi: 10.1016/S0928-8244(03)00228-1 14522453

[54] Kim S, Rajapakse N. Enzymatic production and biological activities of chitosan oligosaccharides (COS): A review. Carbohydrate Polymers. 2005;62(4):357–68. doi: 10.1016/j.carbpol.2005.08.012

